# Out-of-autoclave manufacturing of GLARE panels using resistance heating

**DOI:** 10.1177/0021998317727592

**Published:** 2017-08-31

**Authors:** Bernhard Müller, Genevieve Palardy, Sofia Teixeira De Freitas, Jos Sinke

**Affiliations:** Department of Aerospace Structures and Materials, Faculty of Aerospace Engineering, 2860 Delft University of Technology, The Netherlands

**Keywords:** Out-of-autoclave, resistance heating, fibre metal laminates

## Abstract

Autoclave manufacturing of fibre metal laminates, such as GLARE, is an expensive process. Therefore, there is an increasing interest to find cost-effective out-of-autoclave manufacturing processes without diminishing the laminate quality. The aim of this study is to evaluate the quality of fibre metal laminate panels adhesively bonded and cured using resistance heating. Three manufacturing processes are compared for different layups with an embedded steel mesh at the mid-plane: autoclave curing, resistance bonding of two (autoclave-cured) panels and complete out-of-autoclave resistance curing of panels. Interlaminar shear strength tests and optical microscopy analysis showed that resistance bonding is a promising technique, leading to results comparable to autoclave curing. Resistance curing led to an interlaminar shear strength decrease of 30–60%. A study of the correlation between degree of cure and distance from the mesh revealed the potential of resistance bonding to be used for flexible embedded mesh geometries and on-site repairs.

## Introduction

Fibre metal laminates (FMLs) were developed to reduce the weight and increase the damage tolerance of metallic lightweight structures.^1^ They are composed of alternating metallic sheets and fibre-reinforced epoxy layers.^2^ An FML currently used in the aircraft industry is Glass Laminate Aluminium Reinforced Epoxy, most commonly referred to as GLARE.^3,4^

The main advantage of GLARE, compared to monolithic aluminium structures, is its lower fatigue crack growth rate.^5,6^ In addition, what sets it apart from pure glass fibre laminates is its advanced impact properties,^7^ higher moisture- and UV-resistance,^8^ favourable bearing strength and lightning resistance.^2,4,9^

Currently, autoclave manufacturing is the only process that delivers high quality GLARE panels needed for aerospace applications. However, it is an expensive process, especially when it comes to large parts.^10–12^ Moreover, a second autoclave cycle is often needed to reinforce GLARE panels, for instance in the vicinity of door holes in fuselage panels, in which GLARE doublers or thin aluminium sheets are bonded to the original GLARE fuselage skin.^13^ Apart from the manufacturing costs, previous research has shown that exposing cured GLARE panels to elevated temperatures and thermo-cyclic loads, for example in a second autoclave cycle, can have a detrimental effect on the material properties.^14–20^

Research findings have been reported on out-of-autoclave techniques that can allow localized curing and/or bonding of thermosets. Their common goal is to reduce production costs and focus heating on specific areas. Microwave radiation^21–23^ and induction heating^24–26^ have been investigated to cure or adhesively bond glass and carbon fibre reinforced thermoset composites. The resulting material properties were similar to those obtained with traditional manufacturing techniques, but in some cases, the presence of defects, such as the amount of voids, increased and reached content values up to 20% due to the lower pressure applied during curing.

Another potential localized out-of-autoclave manufacturing technique is resistance heating through the use of a metal mesh embedded at the bondline or in the laminates. This method has been employed extensively to weld thermoplastic composite parts.^27–31^ Those studies demonstrated the potential of resistance heating for joining composites and showed the effect of input parameters, materials and heater mesh on the quality of joints. The same concept has also been investigated to cure thermoset adhesives, resulting in high strength joints with potentially lower manufacturing costs.^32–34^ An important aspect that has been investigated is the identification of processing windows based on input parameters, such as heating elements configuration, to accelerate the curing process with resistance heating.^35,36^

Using the concept of resistance heating to replace the autoclave curing process of GLARE, or to eliminate a second curing cycle when reinforcing GLARE panels, could lead to significant cost reductions. Autoclave manufacturing could be partly replaced by a less expensive, yet more adaptable equipment, consisting mainly of a vacuum bag and a power supply. This high flexibility brings new design opportunities for manufacturing innovative parts, as well as for repair applications. For instance, the location, position and shape of repair patches would be less restricted and on-site repairs using GLARE patches could be made possible. The shape of the resistance heater elements can be customizable and the temperature is generated only where it is required.

The main concern is how the heating elements (or mesh) would affect the quality of the final laminate and how a uniform heating distribution can be guaranteed.

Therefore, the aim of this study is to evaluate the quality of FMLs adhesively bonded or cured using resistance heating.

Three different manufacturing processes are compared: (1) autoclave curing of GLARE panels, (2) resistance bonding of two autoclave-cured GLARE panels and (3) resistance curing of full GLARE panels (complete out-of-autoclave manufacturing). In order to assess the effect of the different manufacturing techniques, a detailed examination of the GLARE panels was carried out based on interlaminar shear strength (ILSS) tests and optical microscopy of the cross-sections and fracture modes.

## Materials

Two types of GLARE laminates were used in this study: GLARE 3-4/3-0.3 and GLARE 5-4/3-0.3. Both laminates consist of four 0.3 mm thick 2024-T3 aluminium layers, bonded together with glass fibre prepregs S2-glass/FM94. The difference between GLARE 3 and GLARE 5 laminates lies in the layup sequence of the prepregs. In GLARE 3, each glass prepreg laminate between the aluminium plates is made of uni-directional (UD) plies with a layup of [0/90]. In GLARE 5, the layup is [0/90/90/0]. The complete layups of GLARE 3-4/3-0.3 and GLARE 5-4/3-0.3 are therefore [Al/0/90/Al/0/90/Al/90/0/Al] and $[\mathrm{Al}/0/90/90/0/\mathrm{Al}{]}_{2s}$, respectively.

Prior to bonding, the aluminium surfaces were pre-treated with chromic acid anodizing and primed with BR 127 (Cytec Engineered Materials, Tempe, Arizona, USA).

The specifications of the stainless steel heater mesh used in this study are listed in Table 1. It has a thickness of 0.8 mm and 200×200meshperlinearinch (25.4 mm).

**Table 1.** Steel heater mesh specifications.^37^

Parameter	Dimension	Unit
Mesh per linear inch	200×200	inch
Thickness	0.8	mm
Wire diameter	0.041	mm
Width of opening	0.089	mm
Open area	46	%
Material	AISI 304L	–

## GLARE panels manufacturing

### Manufacturing methods

#### Reference method: Autoclave curing

**Figure 1.**
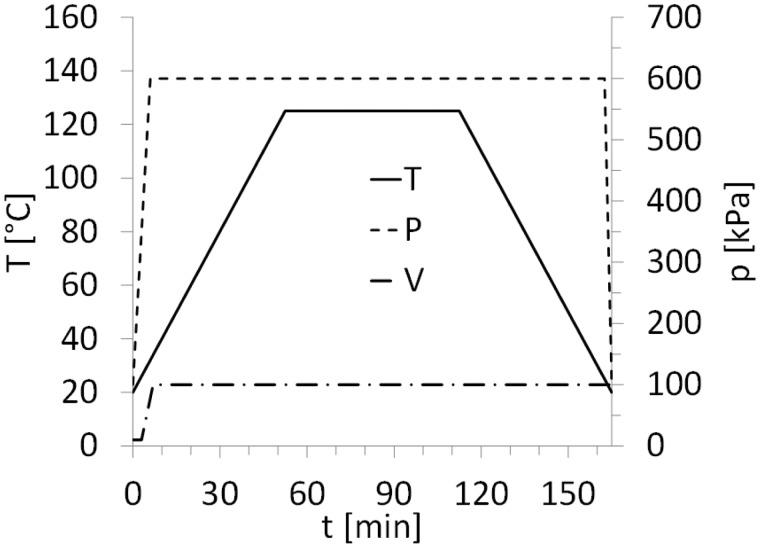
Standard manufacturing conditions for GLARE panels during the cure cycle: Temperature (*T*), pressure in the vacuum bag (*V*) and pressure in the autoclave (*P*).

**Figure 2.**
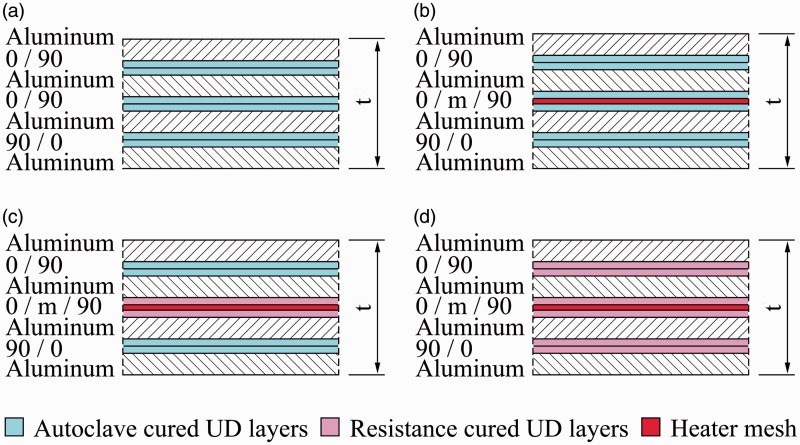
Overview of the investigated manufacturing techniques by means of a GLARE 3-4/3-0.3 layup: Fully autoclave cured (a) without and (b) with mesh, (c) resistance bonded and (d) fully resistance cured.

The standard autoclave cycle for GLARE panels manufacturing is shown in Figure 1. The panels are cured at a temperature (*T*) of 120degC for 1 h, with heating and cooling rates of 2℃/min. The autoclave (*P*) and vacuum bag (*V*) pressures are maintained at 6 bar and 1 bar, respectively. In order to evaluate the effect of the steel mesh on the quality of the panels, independently of the manufacturing process, the autoclave was used to manufacture panels with and without a mesh, as schematically illustrated in Figure 2(a) and (b).

#### Resistance bonding and curing

The work presented in this paper distinguishes between resistance bonding (RB) and resistance curing (RC) of GLARE panels. In the case of the resistance bonding method, firstly, two separate GLARE panels are cured in the autoclave. Secondly, the two panels are brought together with an adhesive layer or glass prepreg layer in between. This layer is subsequently cured using resistance heating (out-of-autoclave secondary bonding), as shown in Figure 2(c). In the case of resistance curing, all prepreg layers through the thickness are cured out-of-autoclave using resistance heating, as shown in Figure 2(d).

During both techniques, a voltage is applied to the metal mesh, which heats up due to its electrical resistance. By following the temperature set points given in the standard autoclave cycle (see Figure 1), it is possible to cure the thermoset layers close to the mesh. Therefore, for both methods, heat is generated from inside the panel, while in autoclave manufacturing, it comes from outside. Another difference compared to autoclave curing is the lower pressure, solely applied with a vacuum bag during the process.

The presence of the epoxy layers ensures electrical isolation between the heater mesh and the aluminium layers. In addition to this, the protective liner which is applied to each single aluminium layer has a very low electrical conductivity. Consequently, the chance of short circuits is reduced during manufacturing.

**Figure 3.**
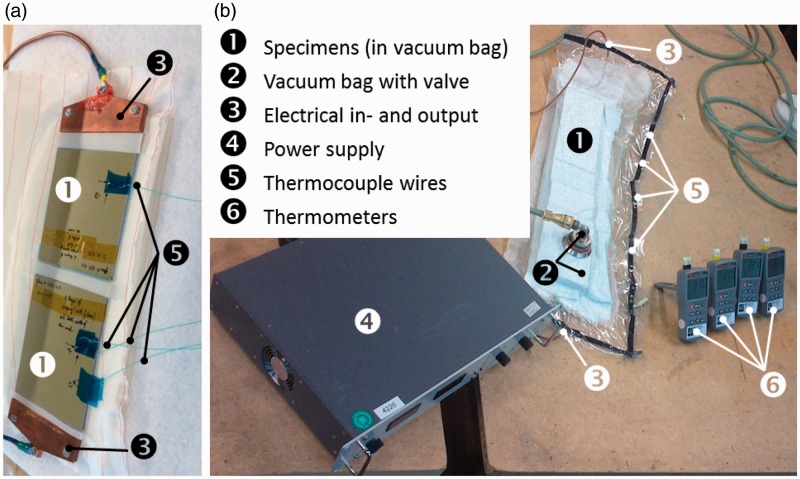
Photo of (a) panels before the curing process and (b) the setup for the out-of-autoclave bonding/curing of GLARE panels.

Figure 3 shows the setup used for resistance bonding and curing of GLARE panels. The main components are (1) the panels, (2) a vacuum bag with a valve, (3) an electrical in- and output, (4) a power supply, (5) four thermocouples and (6) thermometers. Two panels with the same layup were cured simultaneously to reduce manufacturing time and to investigate the possible differences in the process.

The direct voltage (DC) was provided by the power supply and controlled manually in order to follow the temperature set point shown in Figure 1. Three millimeter-thick copper clamps were used for the electrical in- and output to ensure equal distribution of the current. The vacuum bag was used to generate a pressure of one bar. Thermocouples TC I to TC III were embedded in one panel and TC IV, in the other. It was assumed that temperature profiles would be similar in both panels and that therefore, one thermocouple would be sufficient to monitor the process in the second panel.

### GLARE panels layups

Two types of GLARE panels were manufactured: (1) “Full surface mesh”-panels and (2) “Mesh stripe”-panels. In the first, the steel mesh area covers the complete surface area of the GLARE panel. For this type, panels were manufactured using the three different methods mentioned in ‘Manufacturing methods’ section. In the second one, two autoclave cured GLARE panels were bonded using only a mesh stripe, 12.5 mm wide, positioned at the centre of the panels. The aim was to assess the surface area of the embedded mesh needed to guarantee a certain degree of cure.

#### Full surface mesh

**Figure 4.**
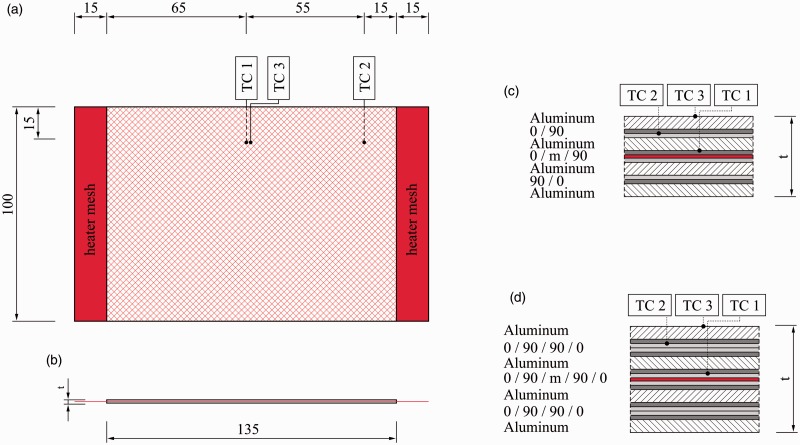
Dimensions of the full surface mesh panels: (a) Top view, (b) side view, details of (c) GLARE 3-4/3-0.3 and (d) GLARE 5-4/3-0.3 cross-sections with an integrated mesh (*m*). Units are in millimetres.

A “Full surface mesh” panel indicates that the embedded mesh covered the entire surface area. In total, eight GLARE 3-4/3-0.3 and eight GLARE 5-4/3-0.3 panels were manufactured according to the layups listed in Tables 2 and 3, respectively. A total of four panels were manufactured with an embedded mesh for each technique: autoclave (A3, A4, A7 and A8), resistance bonding (RB1–RB4) and resistance curing (RC1–RC4). Additionally, four reference samples were cured in the autoclave without mesh (A1, A2, A5 and A6) to investigate its effect on the mechanical performance and quality of the panels. To examine the influence of the glass fibres on the impregnation of the heater mesh, panels with pure epoxy layers adjacent to the mesh were manufactured for the GLARE 3 and GLARE 5 layups (A3, A7, RB1, RB3, RC1 and RC3). Figure 4 depicts the geometry of the panels and the position of the thermocouples during manufacturing of GLARE 3 and GLARE 5.

**Table 2.** Layups for the GLARE 3-4/3-0.3 panels.

Abbr.	Manufacturing Method	Layup
A1	Autoclave	Al/0/90/Al/PE/PE/Al/90/0/Al
A2	Autoclave	Al/0/90/Al/0/90/Al/90/0/Al
A3	Autoclave	Al/0/90/Al/PE/m/PE/Al/90/0/Al
A4	Autoclave	Al/0/90/Al/0/m/90/Al/90/0/Al
RB1	Res. bonding	Al/0/90/Al/PE/m/PE/Al/90/0/Al
RB2	Res. bonding	Al/0/90/Al/0/m/90/Al/90/0/Al
RC1	Res. curing	Al/0/90/Al/PE/m/PE/Al/90/0/Al
RC2	Res. curing	Al/0/90/Al/0/m/90/Al/90/0/Al

Underlined layers indicate they were cured using resistance (res.) heating. (*PE* and *m* are pure epoxy and mesh layers, respectively.)

**Table 3.** Layups for the GLARE 5-4/3-0.3 panels.

Abbr.	Manufacturing Method	Layup
A5	Autoclave	Al/0/90/90/0/Al/PE/PE/PE/PE/Al/0/90/90/0/Al
A6	Autoclave	Al/0/90/90/0/Al/0/90/90/0/Al/0/90/90/0/Al
A7	Autoclave	Al/0/90/90/0/Al/PE/PE/m/PE/PE/ Al/0/90/90/0/Al
A8	Autoclave	Al/0/90/90/0/Al/0/90/m/90/0/Al/0/90/90/0/Al
RB3	Res. bonding	Al/0/90/90/0/Al/PE/PE/m/PE/PE/Al/ 0/90/90/0/Al
RB4	Res. bonding	Al/0/90/90/0/Al/0/90/m/90/0/Al/0/90/90/0/Al
RC3	Res. curing	Al/0/90/90/0/Al/PE/PE/m/PE/PE/ Al/0/90/90/0/Al
RC4	Res. curing	Al/0/90/90/0/Al/0/90/m/90/0/Al/0/90/90/0/Al

Underlined layers indicate they were cured using resistance (res.) heating. (*PE* and *m* are pure epoxy and mesh layers, respectively.)

#### Mesh stripe

**Figure 5.**
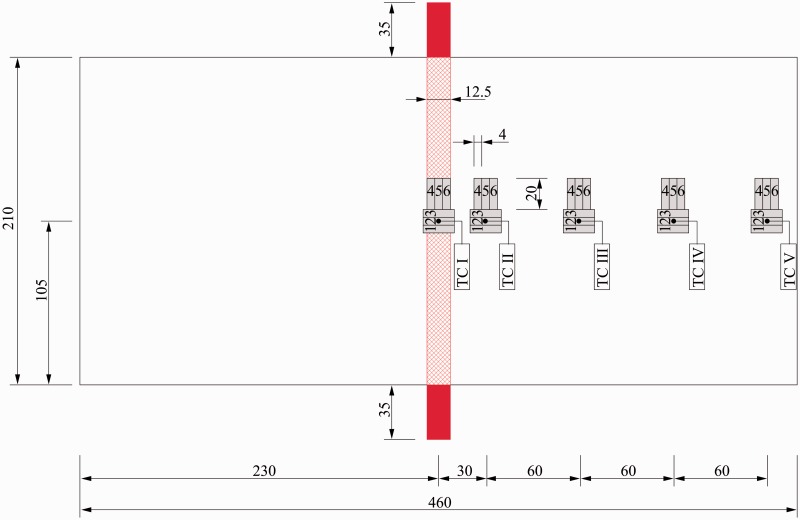
Dimensions of the mesh stripe panel, including the heater mesh (red), the positions of the thermocouples TC I to TC V and the ILSS specimens (grey). Units are in millimeters. ILSS: interlaminar shear strength.

One “mesh stripe” panel was manufactured according to the following procedure: two GLARE 5 panels were first cured in the autoclave, then bonded using resistance heating with a 12.5 mm wide mesh stripe. Figure 5 shows the panel and mesh stripe dimensions, as well as the positions of five thermocouples (TC I to TC V) positioned on the outside surface of the GLARE panels. The layup of the panel is the same as the RB3 panel listed in Table 3.

The electrical current was controlled in such a way that the temperature at the surface of the panel above the mesh was between 120degC and 140degC – controlled by thermocouple I (TC I). This was done in order to increase the overall temperature in the vicinity of the heater mesh to insure a higher degree of cure could be reached.

### Process parameters

#### Full surface mesh

**Figure 6.**
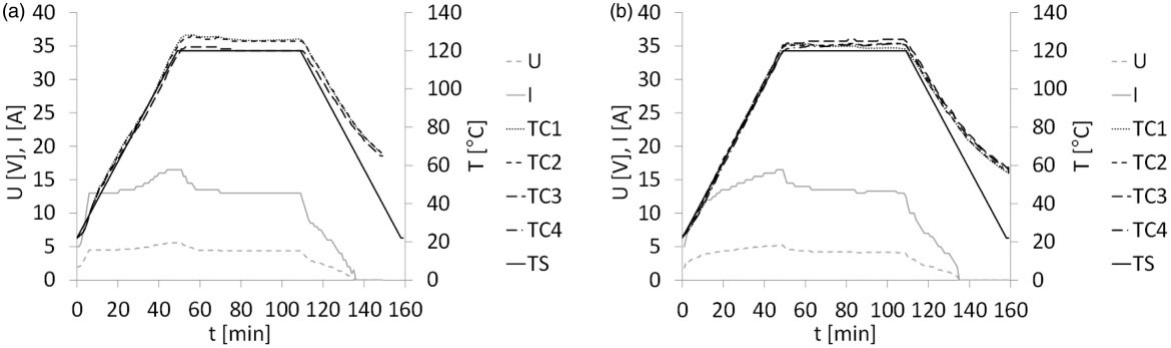
Temperature set point of autoclave cycle (TS), measured temperatures (TC I to TC IV), electrical voltage (U) and current (I) during (a) resistance bonding and (b) resistance curing of GLARE 3 panels with a full surface mesh.

The temperature, electrical voltage and current curves were recorded during the out-of-autoclave manufacturing of GLARE panels using a full surface mesh. A representative example of the curves for resistance bonded GLARE 3 panels is shown in Figure 6(a). The heating ramp rate and hold temperature of the four thermocouples, TC I to TC IV, closely follow the autoclave cycle. The cooling rate, however, slightly deviates from 2℃/min as no external cooling source was used. The electrical voltage was increased and adjusted during the cycle to keep the heating rate and hold temperature as constant as possible.

Representative curves for resistance cured GLARE 3 panels are shown in Figure 6(b). They follow a pattern similar to those for resistance bonded panels. Comparable curves were recorded during the manufacturing of the GLARE 5 panels.

#### Mesh stripe

**Figure 7.**
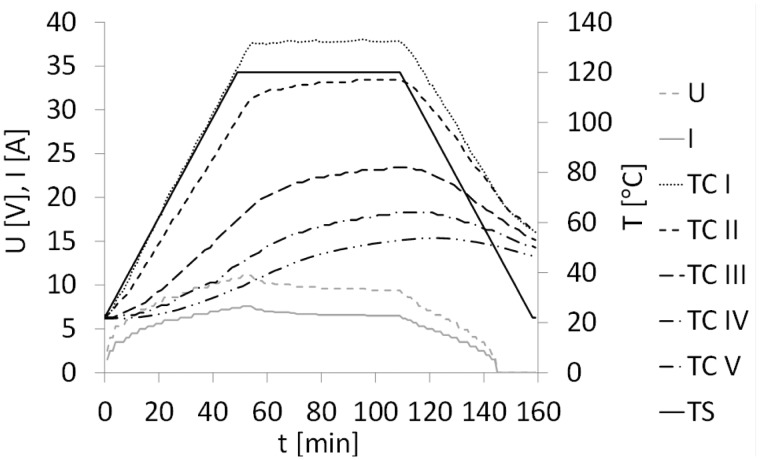
Temperature set point (TS), measured temperatures (TC I to TC V), electrical voltage (U) and current (I) during resistance bonding of the GLARE 5 panels with a mesh stripe.

Figure 7 shows the temperature, electrical voltage and current curves measured during resistance bonding of a GLARE 5 panel using a mesh stripe. The temperature profiles at the locations near the mesh, TC I and TC II, closely followed that of the autoclave cycle (TS). As expected, thermocouples placed further away from the mesh, TC III to TC V, displayed a significant drop in temperature, compared to TC II. The maximum temperature at those locations reached values between 50degC and 80degC.

## Experimental methods

In order to evaluate the performance of the out-of-autoclave manufacturing methods proposed in this work, ILSS tests were performed. It is expected to provide insights into manufacturing quality and the effect of degree of cure on shear strength and adhesion of the epoxy layers.

For each full surface mesh panels – listed in Tables 2 and 3 – six ILSS specimens, 10 mm wide and 20 mm long, were cut from the GLARE panels. Three specimens were tested with the length in the 0deg direction and three specimens in the 90deg direction.

In the case of the mesh stripe panel, a total of six ILSS specimens in the 0deg and 90deg directions were tested for each thermocouple position in order to investigate the correlation between the distance from the mesh and the resulting effect on the ILSS values (see positions in Figure 5). The specimen dimensions were 4mm×20mm to focus more specifically on locations where different degrees of cure were expected.

**Figure 8.**
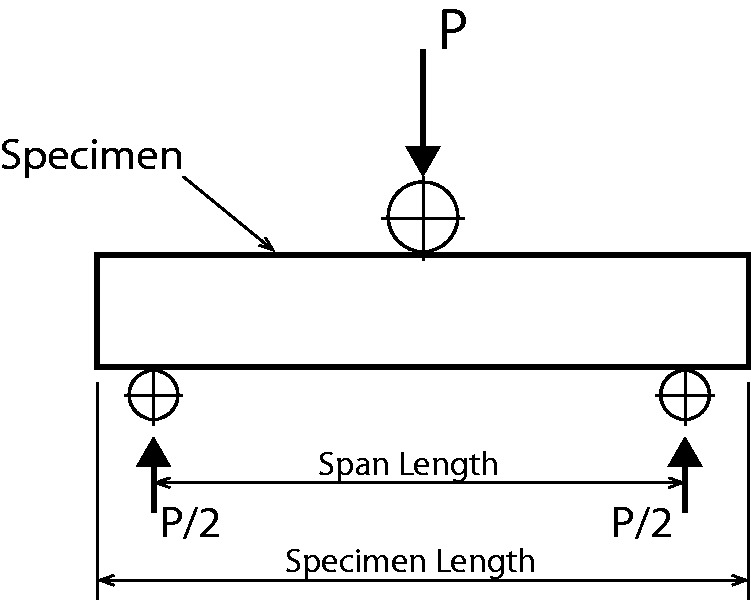
Schematic representation of the ILSS setup.^38^ ILSS: interlaminar shear strength.

The ILSS tests were performed according to the ASTM D2344 standard for short-beam strength of polymer matrix composite materials and their laminates.^38^ A schematic figure of the setup is given in Figure 8. The loading span length-to-specimen thickness ratio was kept to 4.0 as recommended by the ASTM standard. In both cases, all ILSS tests were conducted on a 25 kN press with a test speed of 1 mm/min. During tests, the load-displacement curves were recorded. After testing, the failure mode of the ILSS specimens was examined with a high-resolution Keyence stereomicroscope. Furthermore, the manufacturing quality of the panels was assessed through cross-sectional microscopy.

## Experimental results

### Full surface mesh panels

#### Mechanical performance

**Figure 9.**
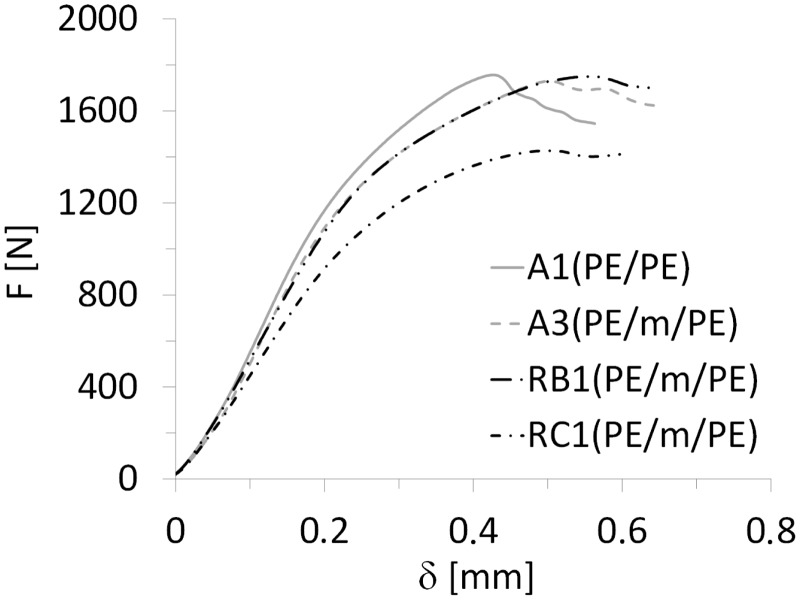
Typical force-displacement curves of ILSS tests on GLARE 3 specimens manufactured by autoclave, resistance bonding and resistance curing when using a full surface mesh. ILSS: interlaminar shear strength.

Figure 9 shows representative force-displacement (F-δ) curves of the ILSS tests for GLARE 3 samples manufactured by all three methods described in ‘Manufacturing methods’ section – for the complete layup of the panels, please see Tables 2 and 3. The autoclave specimens manufactured without a mesh (A1) displayed the steepest slope, followed by a sharp decrease in the load when fracture occurred. The slope of the curves, proportional to the stiffness of the specimens, slightly decreased when a mesh was placed at the interface (A3 and RB1). For A3 and RB1 layups, the curves followed a similar trend and reached a maximum force close to A1, but at a higher displacement value. The RC1 layup deviated from the other samples and presented a lower stiffness and maximum load.

**Figure 10.**
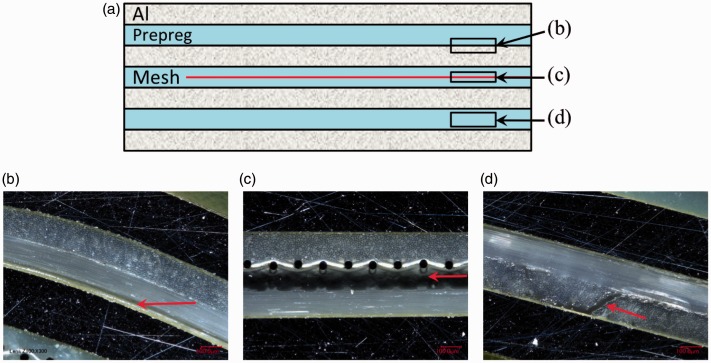
Typical failure modes: (a) Schematic GLARE cross-section with failure mode locations (red arrows) and representative cross-sectional microscopy images of (b) intralaminar failure in prepreg layer close to aluminium layer, (c) failure at the mesh interface, (d) intralaminar failure in the outer prepreg layers. Scale: 100μm.

Figure 10(a) schematically depicts the main failure modes observed in ILSS specimens for GLARE 3 panels. Intralaminar failure in the prepreg layer, close to the aluminium layer (Figure 10(b)) mainly occurred for autoclave-cured samples without and with a mesh, A1 to A4 (Table 2), as well as for resistance bonded specimens with pure epoxy layers at the mesh (RB1). On the other hand, failure at the mesh interface (Figure 10(c)) was only observed for resistance bonded panels when prepreg layers were placed at the interface (RB2 layup). For resistance cured specimens (RC1 and RC2), fracture took place in the outer prepreg layers, as shown in Figure 10(d). It is to be noted that similar failure modes were found for GLARE 5 samples.

**Figure 11.**
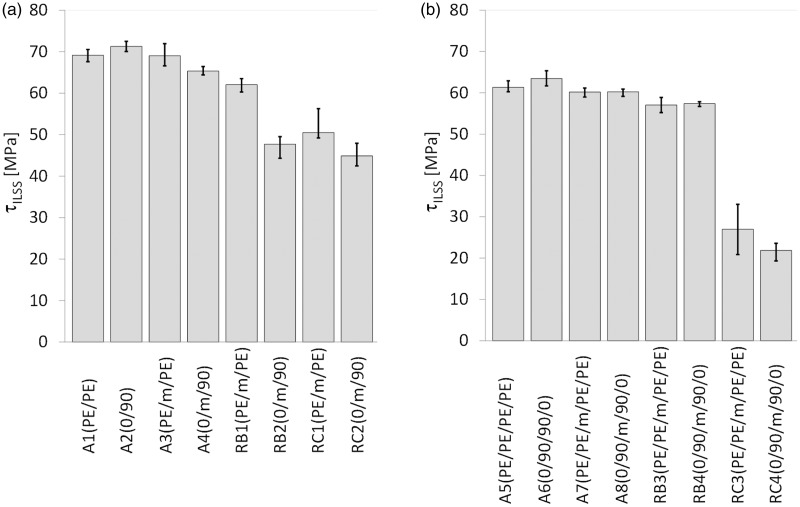
Average ILSS values for (a) GLARE 3 and (b) GLARE 5 specimens manufactured by autoclave, resistance bonding and resistance curing, according to the layups listed in Tables 2 and 3. The error bars show the scatter range with minimum and maximum ILSS values for each group of specimens. ILSS: interlaminar shear strength.

The ILSS was calculated based on the maximum force measured in the force-displacement curves (Figure 9), as given by the ASTM D2344 standard:

(1) $$\tau_{\mathrm{ILSS}}=\frac{0.75F_{\text{max}}}{\mathrm{WL}}$$

where *F_max_* is the maximum load, and *W* and *L* are the width and length of the specimen, respectively. Figure 11 and Table 4 summarize the ILSS values for (a) GLARE 3 and (b) GLARE 5 specimens manufactured by autoclave, resistance bonding and resistance curing methods. The figure shows the average values and the scatter range of the five ILSS tests conducted for each configuration as listed in Tables 2 and 3.

**Table 4.** Average ILSS values *τ_ILSS_* for the GLARE 3 (GL3) and GLARE 5 (GL5) specimens.

GL3	*τ_ILSS_* (MPa)	GL5	*τ_ILSS_* (MPa)
A1	69.2	A5	61.3
A2	71.3	A6	63.5
A3	69.1	A7	60.2
A4	65.3	A8	60.3
RB1	62.1	RB3	57.1
RB2	47.7	RB4	57.4
RC1	50.5	RC3	27.0
RC2	44.9	RC4	21.9

ILSS: interlaminar shear strength.

For both GLARE 3 and GLARE 5 samples manufactured in the autoclave (A1 to A8), the heater mesh did not have a significant effect on the ILSS values, remaining within scatter range. Resistance bonded specimens (RB1 to RB4) displayed similar shear strength values to the autoclave panels, with the exception of RB2, which dropped to 47.7 MPa. This is consistent with the failure mode presented in Figure 10(c), which is located at the mesh interface, likely due to poor resin impregnation because of the prepreg layers. When the panels were resistance cured, their average ILSS decreased by 27% to 31% for RC1 and RC2, and by 55% to 64% for RC3 and RC4, with comparison to the panels manufactured by autoclave with a mesh.

#### Optical microscopy analysis

**Figure 12.**
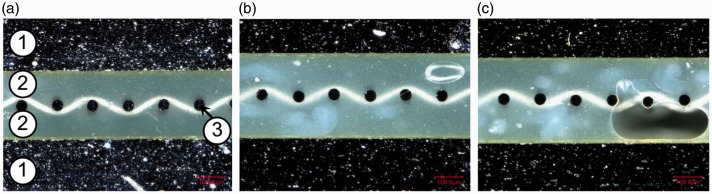
Cross-sectional microscopy images of GLARE 3 panels with embedded heater mesh between pure epoxy layers: (a) Autoclave manufacturing, (b) resistance bonding and (c) resistance curing. Legend: (1) aluminium layers, (2) pure epoxy layers and (3) heater mesh. Scale: 100μm.

**Figure 13.**
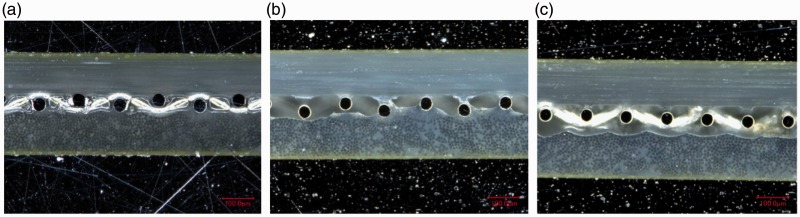
Cross-sectional microscopy images of GLARE 3 panels with embedded heater mesh between prepreg layers: (a) autoclave manufacturing, (b) resistance bonding and (c) resistance curing. Scale: 100μm.

Cross-sections of the panels manufactured according to the methods and layups presented in Tables 2 and 3 were observed by optical microscopy to provide insight regarding the mechanical performance presented in ‘Mechanical performance’ section (below the ‘Full surface mesh panels’ section). Figure 12 shows representative images of GLARE 3 panels manufactured by all three methods and compares the heater mesh impregnation when using pure epoxy layers as the middle plies (A3, RB1 and RC1). Autoclave cured panels (Figure 12(a)) exhibited the highest quality of mesh impregnation and the thinnest resin layer due to the higher pressure applied during manufacturing. It was observed that the presence of voids at the interface generally increased from resistance bonded (Figure 12(b)) to resistance cured (Figure 12(c)) panels. For the layups using prepreg layers only (A4, RB2 and RC2), the mesh impregnation significantly decreased compared to the use of pure epoxy layers, due to the lower resin content (Figure 13). Similarly to Figure 12, the presence of voids increased from autoclave (Figure 13(a)), to resistance bonded (Figure 13(b)), to resistance cured (Figure 13(c)) panels. For the latter, a clear gap between the layers on both sides of the mesh was noticed.

**Figure 14.**
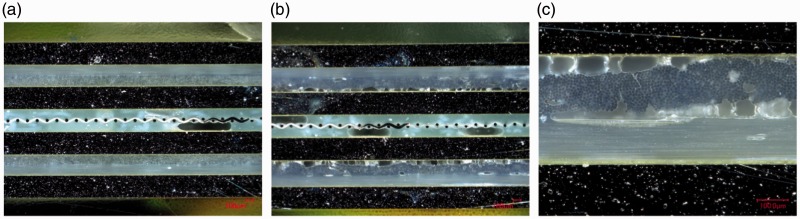
Cross-sectional microscopy images of GLARE 3 panels comparing the quality of the outer prepreg layers: (a) Resistance bonded panel, (b) resistance cured panel and (c) higher magnification image of bottom plies in (b). Scale: 100μm.

The quality of the outer prepreg layers for GLARE 3 specimens manufactured by resistance bonding and curing is compared on Figure 14(a) and (b), respectively. For resistance cured panels, several voids are present, especially at the aluminium-prepreg interface (Figure 14(c)), possibly as a result of the lower pressure applied during out-of-autoclave manufacturing.

These observations can explain the failure modes witnessed in Figure 10. For resistance bonded specimens with prepreg layers (RB2), failure occurred at the mesh interface because of poor impregnation. The use of pure epoxy layers in the RB1 layup eliminated this weakness and therefore, this resulted into intralaminar failure, as seen in Figure 10(b). For resistance cured samples, fracture was noted in the outer prepreg layers, likely due to their lower quality compared to the mesh impregnation. It is also possible that residual stress concentrations developed during the curing process may have contributed to crack initiation.

**Figure 15.**
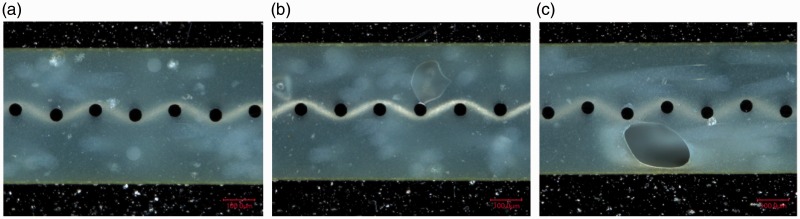
Cross-sectional microscopy images of GLARE 5 panels with embedded heater mesh with four pure resin layers: (a) Autoclave manufacturing, (b) resistance bonding and (c) resistance curing. Scale: 100μm.

For GLARE 5 panels, the use of four pure resin layers at the mesh interface (Figure 15) led to comparable impregnation to the GLARE 3 specimens (Figure 12). It can be inferred that using only two pure epoxy layers are sufficient for proper impregnation and quality.

### Mesh stripe panel

#### Mechanical performance

**Figure 16.**
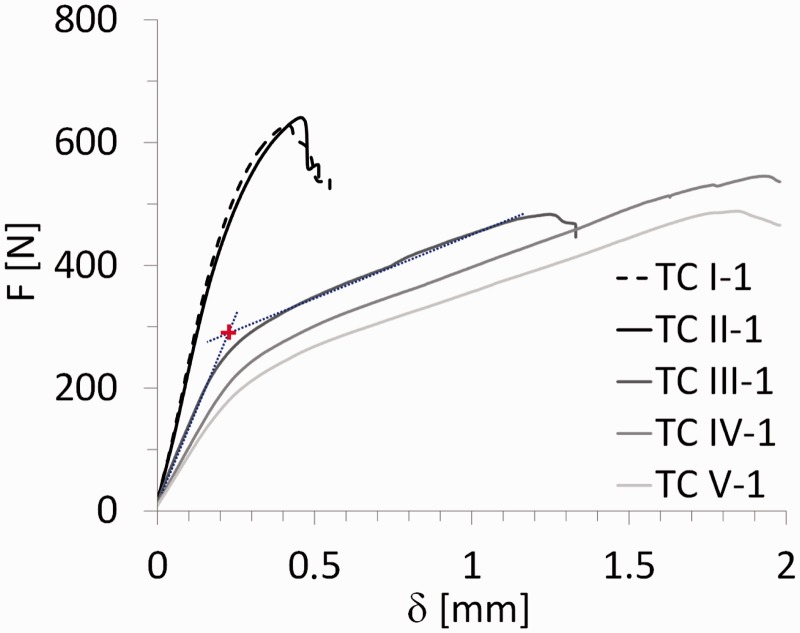
Representative force-displacement curves of ILSS tests on mesh stripe specimens – ‘+’ represents the bilinear intersection of the slopes. ILSS: interlaminar shear strength.

Figure 16 shows representative F-δ curves of the ILSS tests at the five (thermocouple) positions (see Figure 5). The ILSS specimens for positions TC I and TC II display the steepest F-δ curve slopes, followed by a drop in the load after failure. These positions also display the highest maximum load when compared to the remaining positions (TC III, TC IV and TC V).

The F-δ curves of the ILSS specimens from the positions TC III, TC IV and TC V are significantly different. They approximate to a bi-linear behaviour – see Figure 16 for TC III-1. The initial slope is lower than for positions TC I and TC II. After this initial slope, a significant plastic deformation plateau is followed before final failure.

Although also present, the change of slope and the plastic deformation in positions TC I and TC II is almost insignificant when compared with positions TC III, TC IV and TC V.

As for the failure modes, positions TC I and TC II fail similarly as the specimens for full surface mesh resistance bonding using pure epoxy (RB1 and RB3): intralaminar failure in the prepreg layer close to the aluminium layer (Figure 10(b)). This indicates a good adhesion on the curing process of the resistance bonded layers. In fact, the F-δ curves of positions TC I and TC II are more comparable with the ones presented for the full surface mesh specimens in Figure 9 than with the positions TC III to TC V.

**Figure 17.**
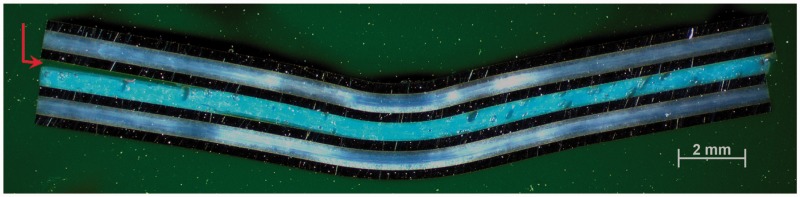
Cross-sectional microscopy image of a representative ILSS specimen at locations TC III, TC IV and TC V. The red arrow indicates the crack initiation. ILSS: interlaminar shear strength.

The failure mechanism was significantly different for positions TC III, TC IV and TC V. Figure 17 shows the typical failure mode of these specimens. The final failure typically occurred at the interface between the pure epoxy layers and the adjacent aluminium layers. This indicates a poor adhesion quality during the curing process of those layers. In addition to this, a significant permanent plastic deformation can be observed after failure.

This interface failure justifies the different F-δ behaviour of the specimens at positions TC III, TC IV and TC V when compared to TC I and TC II. The (not-fully-cured) pure epoxy layer could not take significant longitudinal shear stress and therefore could not guarantee the continuous strain distribution through the laminate thickness. This discontinuity in strains results in significantly higher normal stresses at the aluminium layers when compared to the situation of continuous longitudinal strains through the laminate thickness for the same load – as in the case of positions TC I and TC II. Therefore, the aluminium layers yield at mid span at a much lower load level for positions TC III, TC IV and TC V, as seen in Figure 16. The displacement plateau shown at these curves corresponds probably to the aluminium ductility after yield.

**Figure 18.**
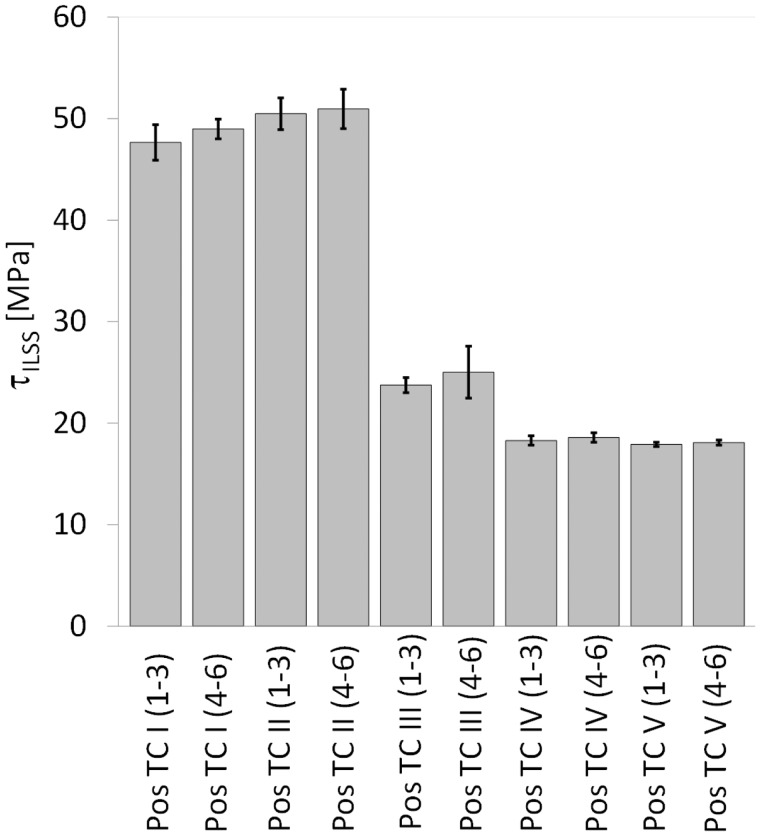
Average ILSS values *τ_ILSS_* at the positions indicated in Figure 5. The error bars show the scatter range with minimum and maximum ILSS values for each group of specimens. ILSS: interlaminar shear strength.

Figure 18 and Table 5 show the average ILSS values for the five positions, both longitudinal direction (specimens 1 to 3) and transverse direction (specimens 4 to 6). For positions TC I and TC II, the ILSS values were determined using the maximum load value, as was the case for full surface mesh samples (‘Mechanical performance’ section (below the ‘Full surface mesh panels’ section)). For positions TC III, TC IV and TC V, the ILSS values were determined using a bilinear intersection – marked as ‘+’ in Figure 16. There are two main reasons to use the intersection values for the latter positions. Firstly, the F-δ curve and the failure mechanics show that the aluminium starts to yield at the onset load values. This is considered to be the failure of the specimens for position TC III to TC V. Secondly, the ILSS formula shown in ‘Full surface mesh panels’ section is only valid in the linear elastic regime. The maximum load of positions TC III to TC V occurs after significant plastic deformation and therefore, the formula is no longer valid.

**Table 5.** Average ILSS for GLARE 5 specimens manufactured by resistance bonding with a mesh stripe.

Position	Unit	TC I	TC II	TC III	TC IV	TC V
*τ_ILSS_* (1–3)	(N/mm^2^)	47.6	50.5	23.7	18.3	17.9
*τ_ILSS_* (4–6)	(N/mm^2^)	49.0	50.9	25.0	18.6	18.1

ILSS: interlaminar shear strength.

The average ILSS value of position TC I (1–3), where the mesh stripe was located, was 47.6 MPa. Specimens adjacent to the mesh, TC II (1–3) – 30 mm from the centre of the mesh, had similar ILSS values (50.5 MPa). At distances farther away from the mesh, the average shear strength decreases significantly at 90 mm distance by 50% (TC III) and from 150 mm on by 60% (TC IV and TC V). ILSS specimens tested in the transverse direction showed similar shear strength values as in the longitudinal direction.

The ILSS values of positions TC I and TC II (47.6 to 50.9 MPa) are of the same order as the one obtained for RB3 specimens (see Table 4, 57.1 MPa). Both have the same layup. The significant decrease in ILSS values for positions TC III to TC V is related with the different bending behaviour shown by the F-δ curve and failure mechanics (significant yield of the aluminium before debonding of the aluminium layers), likely due to low degree of cure.

#### Optical microscopy analysis

**Figure 19.**
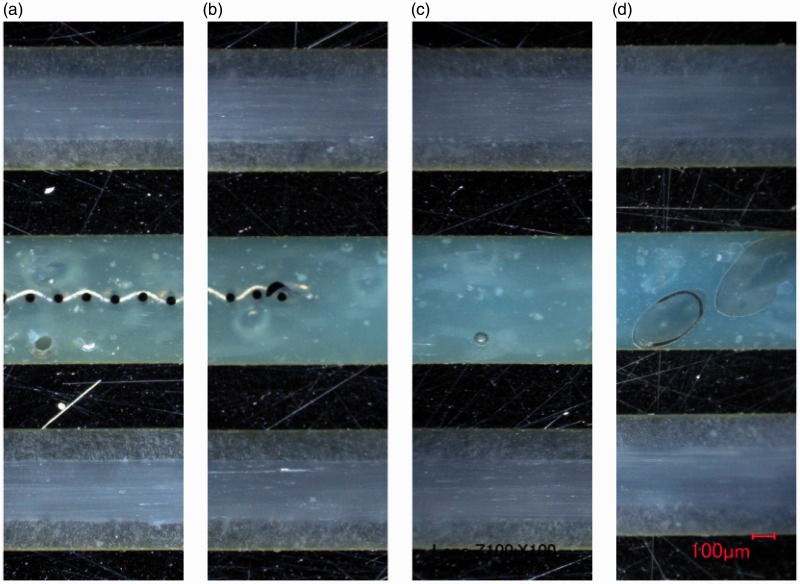
Cross-sectional microscopy images of GLARE 5 panels resistance bonded with a mesh stripe at different locations from the mesh: (a) TC I, (b) Mesh transition between TC I and TC II, (c) TC II and (d) TC III. Scale: 100μm.

In order to assess the mesh impregnation quality and explain the results presented in ‘Mechanical performance’ section (below the ‘Mesh stripe panel’ section), cross-sections were observed by optical microscopy, as was the case for full surface mesh panels. Figure 19 shows cross-sectional images of the panel manufactured by resistance bonding. Locations TC I to TC III, based on Figure 5, are shown from (a) to (d). Good mesh impregnation was observed, even at the transition from TC I to TC II. For location TC III, the presence of large voids in the pure epoxy layers was significant. These voids were also observed at locations further away from the mesh, TC IV to TC V.

These observations can justify and support the significant difference in the mechanical behaviour of the specimens close to the mesh – Positions TC I and TC II, and far from the mesh – Positions TC III, TC IV and TC V. The large voids observed in the latter confirm the poor manufacturing quality and corresponding poor mechanical performance observed at those locations.

## Discussion

### Comparison: Autoclave – Resistance bonding – Resistance curing

Based on the ILSS and microscopy results presented in ‘Full surface mesh panels’ section for the autoclave cured, resistance bonded and resistance cured specimens, three main observations can be highlighted.

Firstly, for the autoclave cured specimens, there were minor to no changes in the quasi-static behaviour and in the cross-section quality without (A1, A2, A5 and A6) and with (A3, A4, A7 and A8) an embedded stainless steel mesh (see Figures 9 and 11). The most significant difference was noted when comparing the GLARE 3 panels without (A2) and with (A4) an embedded heater mesh when prepreg layers were placed adjacent to the mesh. This was the result of poorer impregnation of the mesh due to lower epoxy volume content (see Figures 12 and 13).

Secondly, the ILSS values, failure modes and corresponding cross-section quality were comparable for the autoclave cured and resistance bonded GLARE 3 and GLARE 5 panels. The exception which did not follow this trend was, similarly to the autoclave cured panels, the resistance bonded GLARE 3 panel with prepreg layers adjacent to the mesh (RB2 panel). It is assumed that the epoxy volume content was not sufficient to impregnate the heater mesh properly. Therefore, the crack initiated at the epoxy-heater mesh interface for RB2 specimens (Figure 10(c)).

Finally, the resistance curing method produced panels of distinctively lower quality with an increased presence of voids in all prepreg layers (including the ones adjacent to the heater mesh). This led to a decrease in the ILSS values and the onset of failure in the outer prepreg layers. As voids disrupt the homogeneity of the material and act as crack initiators, a higher void content consequently increases the chance of failure at lower stress values and thus, leads to a decrease of the (static) strength. However, this behaviour was more noticeable for the GLARE 5 specimens, compared to GLARE 3, as the void content was likely higher with a lower aluminium surface area over the cross-section (see ‘Materials’ section).

### Degree of cure vs ILSS – Resistance bonding with mesh stripe

Using a mesh stripe instead of a full surface mesh for resistance bonding of GLARE panels severely affects the temperature distribution (see Figure 7). Thus, the aim of this study was to monitor the in-plane temperature distribution during resistance bonding to investigate its effect on the degree of cure and ILSS values at different positions from the mesh (Figure 5).

**Figure 20.**
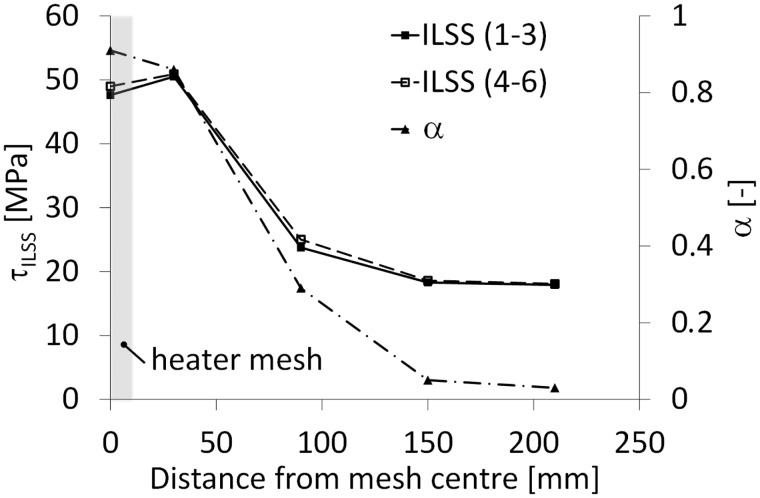
Average ILSS values (*τ_ILSS_*) and estimated degree of cure, *α*, at different positions from the heater mesh stripe (based on Figure 5). ILSS: interlaminar shear strength.

As previously presented in Figure 18, reasonable ILSS values were determined at locations TC I and TC II, corresponding to distances of up to 30 mm from the heater mesh. Knowing the temperature profiles at different positions (Figure 7), the degree of cure, *α*, can be estimated from TC I to TC V based on Kamal–Sourour’s cure kinetics model presented in Abouhamzeh et al.^39^ In order to do so, three main assumptions were made. Firstly, the same heating/cooling rate for all positions as the one used in the standard manufacturing cycle was assumed (±2degC). Secondly, the maximum temperature at each position remained constant for 60 min. Finally, these constant temperature values for TC I to TC V were assumed to be equal to 130degC,120degC,80degC,60degC and 50degC. The expected degree of cure is plotted in Figure 20, along with the corresponding average ILSS values, *τ_ILSS_*, as shown in ‘Mechanical performance’ section (below the ‘Mesh stripe panel’ section).

For both cases, as the distance from the mesh increases, the degree of cure and ILSS values significantly drop between 30 mm (TC II) and 90 mm (TC III), which is consistent with literature on epoxy/glass fibre systems submitted to different cure cycles.^40^ These findings suggest that using a spacing of approximately 60 mm between mesh stripes would allow to maintain reasonable degree of cure and manufacturing quality. This can provide flexibility in the case where a more complex mesh geometry might be required depending on the parts to be resistance bonded.

## Conclusions

Three manufacturing techniques for GLARE panels were investigated and compared: full autoclave curing, resistance bonding of two autoclave-cured panels and complete out-of-autoclave resistance curing. For the latter two methods, a steel mesh was placed at the panels’ mid-plane for bonding or curing through resistance heating. The effect of the heater element was investigated as a first step for autoclave cured panels. No major differences in the static behaviour and manufacturing quality were found between panels with and without an embedded heater mesh.

The comparison of the different manufacturing techniques and layups with an embedded steel mesh across the whole surface at the mid-plane showed that resistance bonding is a promising technique, which leads to comparable ILSS values to the fully autoclave cured samples with a maximum decrease of 10%. Resistance cured samples, however, do not show sufficient manufacturing quality. The significant presence of voids leads to a decrease of the ILSS values, especially for the GLARE 5 samples. In all cases, the importance of a proper mesh impregnation was noted. The best quality was obtained with pure epoxy layers at the mesh interface, while the use of only one prepreg layer on each side of the mesh was more likely to promote crack initiation.

As a first step toward a flexible heater mesh geometry, two GLARE 5 panels were resistance bonded using a 12.5 mm wide (stripe) heater element. The study showed that the degree of cure and ILSS values at distances larger than 30 mm from the mesh decreased significantly. This suggests that a spacing of 60 mm between mesh stripes would allow to maintain high quality and decrease energy consumption during manufacturing. Further investigation into customisable mesh dimensions for flexible on-site repairs could be a focus of future research.

The promising results obtained for the resistance bonded panels with an embedded mesh across the full surface demonstrated the capability to accomplish comparable quality to autoclave manufacturing with minimal equipment (vacuum bag, power supply and thermocouples). Hence, this flexible technique could eliminate a second costly autoclave cycle in the case where, for instance, doublers or stringers need to be bonded to GLARE panels. Furthermore, it can be used for assembly of larger GLARE panels through e.g. resistance bonded scarf joints.
